# A method to increase reproducibility in adult ventricular myocyte sizing and flow cytometry: Avoiding cell size bias in single cell preparations

**DOI:** 10.1371/journal.pone.0186792

**Published:** 2017-10-30

**Authors:** Javier E. López, Katrin Jaradeh, Emmanuel Silva, Shadi Aminololama-Shakeri, Paul C. Simpson

**Affiliations:** 1 University of California, Davis, CA, United States of America; 2 VA Medical Center and University of California, San Francisco, CA, United States of America; Rutgers New Jersey Medical School, UNITED STATES

## Abstract

**Rationale:**

Flow cytometry (FCM) of ventricular myocytes (VMs) is an emerging technology in adult cardiac research that is challenged by the wide variety of VM shapes and sizes. Cellular variability and cytometer flow cell size can affect cytometer performance. These two factors of variance limit assay validity and reproducibility across laboratories. Washing and filtering of ventricular cells in suspension are routinely done to prevent cell clumping and minimize data variability without the appropriate standardization. We hypothesize that washing and filtering arbitrarily biases towards sampling smaller VMs than what actually exist in the adult heart.

**Objective:**

To determine the impact of washing and filtering on adult ventricular cells for cell sizing and FCM.

**Methods and results:**

Left ventricular cardiac cells in single-cell suspension were harvested from New Zealand White rabbits and fixed prior to analysis. Each ventricular sample was aliquoted before washing or filtering through a 40, 70, 100 or 200μm mesh. The outcomes of the study are VM volume by Coulter Multisizer and light-scatter signatures by FCM. Data are presented as mean±SD.

Myocyte volumes without washing or filtering (NF) served as the “gold standard” within the sample and ranged from 11,017 to 46,926μm^3^. Filtering each animal sample through a 200μm mesh caused no variation in the post-filtration volume (1.01+0.01 fold vs. NF, n = 4 rabbits, *p* = 0.999) with an intra-assay coefficient of variation (%CV) of <5% for all 4 samples. Filtering each sample through a 40, 70 or 100μm mesh invariably reduced the post-filtration volume by 41±10%, 9.0±0.8% and 8.8±0.8% respectively (n = 4 rabbits, *p*<0.0001), and increased the %CV (18% to 1.3%). The high light-scatter signature by FCM, a simple parameter for the identification of ventricular myocytes, was measured after washing and filtering. Washing discarded VMs and filtering cells through a 40 or 100μm mesh reduced larger VM by 46% or 11% respectively (n = 6 from 2 rabbits, *p*<0.001).

**Conclusion:**

Washing and filtering VM suspensions through meshes 100μm or less biases myocyte volumes to smaller sizes, excludes larger cells, and increases VM variability. These findings indicate that validity and reproducibility across laboratories can be compromised unless cell preparation is standardized. We propose no wash prior to fixation and a 200μm mesh for filtrations to provide a reproducible standard for VM studies using FCM.

## Introduction

To study cellular mechanisms of normal cardiac function and pathology, high-quality isolated adult myocytes are essential.[1, 2] Reports on cell-to-cell imbalances in gene expression[3–5], heterogeneous expression of sarcomeric proteins[6–10], and adrenergic receptors[11] in myocyte sub-populations underscore the importance of single-cell analysis to understand cardiac biology. More broadly, single-cell analysis of all individual cells present in a heart is of high impact[12] and on par with the goals of the NIH Human BioMolecular Atlas Program.[13] This program seeks to facilitate the mapping of all single cells within tissues to understand the relationship between cellular organization and organ function.

Flow cytometry (FCM) is a powerful tool for phenotyping individual cells within complex cell populations. In cardiac biology, the use of FCM in adult ventricular myocytes (VMs) isn’t frequently implemented in experiments due to the biophysical challenges caused by large, elongated, and irregularly shaped VMs.[6, 10] In 1979, Nash and colleagues[14] reported the first evaluation of VM size and shape using a Coulter Counter Model B and a Coulter-Telefunken focused-flow system both fitted with a 200μm aperture. They reported the impact of aligning rod-shaped VMs with flow direction along a confined path: “Unfortunately this instrument tends to become blocked rather easily when used with heart muscle cells.”[14]

The next milestone in VM FCM came in 1998, when Diez and Simm[15] reported the first successful fluorescent-activated cell sort (FACS) to purify rod-shaped rat VMs. In this study, cell preparations were filtered through a 250μm mesh, washed with low-speed centrifugation (25g x 3 min) and fixed with ethanol for RNA preservation. VMs were sorted through an Epics Elite ESP flow cytometer fitted with a 100μm sort tip. In 2017[10], we showed that mouse VMs fixed with paraformaldehyde (PFA) for FACS could not be easily purified in a BD Influx sorter fitted with a standard 100μm nozzle due to clogging in the instrument, as reported by Nash and colleagues[14]. Ultimately, a new large-particle set up with a 200μm nozzle was needed to overcome the biophysical challenge of purifying this large cell type.[10]

Limiting instrument clogs is critical to obtain reproducible data in standard flow cytometry.[6] Generally, investigators new to FCM rely on core facilities to provide standard protocols for sample preparation. Most of these protocols have been developed for optimal results in round-shaped cells of <30μm in diameter (e.g. nucleated blood cells, culture cell lines, etc.). The size of adult VMs naturally ranges from 90–130μm in length and 20–50μm in width.[10] The flow cell of most flow cytometers range in size from 50μm to 200μm. FCM analyzers unlike cell sorters usually have a fixed flow cell that cannot be modified.[16] Under normal hydrodynamic conditions, the cell stream is optimally focused down to ~1/5 of the flow cell for optimal performance. The relationship of cell sizes to the flow cell present a biophysical challenge when generating a stable stream of larger VMs along the flow direction. Maximizing the ratio of the cell stream (containing the VMs) to the flow cell size is critical for maximizing signal reliability and minimizing clogging of the instrument.[10, 17] The initial response to a cytometer-to-cell size mismatch or a clogged instrument is to usually add additional washes and/or filter the preparation through smaller meshes to improve performance. Although these protocol modifications can enhance reliability and precision, their effect on accuracy are usually unknown. Because VMs FCM is still in the early stages of implementation across many laboratories, validated standards for reliable preparations are not yet developed.

We hypothesize that low-speed centrifugation for cell washes and variable filtering mesh sizes arbitrarily alter myocyte sampling independent of biology. We aimed to define method variations that minimize altering the experimental samples when compared to the parent cell population. Our results uncovered a tendency to sample fewer and smaller VMs than actually exist in the adult heart and provide guidance on how to standardize these preparations to minimize this variability.

## Materials and methods

### Animal samples

The animal care and experimental protocols followed the US National Institutes of Health guidelines and were approved by the Institutional Animal Care and Use Committees of the University of California at Davis. Ventricular myocyte hypertrophy (HT) was induced in New Zealand White rabbits by combining aortic valve insufficiency and descending aorta banding as described.[18]

### Single cell preparations and filtering

Single cell dispersions were carried out by coronary perfusion and enzymatic digestion as reported[18]. To represent all ventricular cells harvested from the left ventricle, the parent cell suspension is not washed before fixing unless otherwise noted. Cells were fixed in 15ml polypropylene conical tubes for 10min at RT by gently mixing cells in a 10-15mls suspension with PFA at a final concentration of 0.4%; this avoids cell clumping.

Prior to testing the effect of gentle centrifugation (i.e. low-speed washes) and/or filtering as experimental variables, fixed cells in suspension were centrifuged at 800xg for 5min at RT, and washed once with 2mls of filter-sterilized (0.2μm) calcium and magnesium free PBS (CMF-PBS). This wash does not change the morphology or composition of the parent cell suspension but removes traces of PFA[10].

To test the impact of low-speed washes on cell composition, the parent cell suspension was pelleted at low speed twice (50xg for 1 min at RT x2), and supernatants were pooled before FCM analysis. This experimental wash is usually done to generate a pellet with large cells (i.e. VM) and a supernatant with small cells (i.e. nonmyocytes). For filtering analysis, fixed cells in suspension were passed through a 40, 70, 100 or 200μm mesh and washed with an equal volume of CMF-PBS prior to cell sizing or FCM. All cells tested by FCM had to be filtered because not-filtered cells cause clogging of the cytometer.

### Myocyte sizing

The median volumes of 4,000–7,000 VMs were measured with a Coulter Multisizer 4 (Beckman Coulter, Brea, CA).[10]

### Flow cytometry

Fixed ventricular cells in suspension were treated with DNAse-free RNAse A 2μg/ml (Sigma, St. Louis, MO) and labeled with propidium iodide (PI, BD Pharmingen) prior to flow cytometry to detect nucleated particles as described[6, 19, 20]. Cells from pellet, supernatant (i.e. post-spin) and parent suspension (i.e. pre-spin) were analyzed for light-scatter signatures using a standard FACScan (BD Biosciences, San Jose, CA) with a standard 430μm x 180μm flow cell as described.[6, 19, 20] Side light-scatter is validated as a size indicator of large myocytes.[6]

Myocytes were labeled with mouse anti-beta-myosin heavy chain (β-MyHC) IgG (NOQ7.5.4D, Sigma-Aldrich, St Louis, MO) using a Zenon kit (Invitrogen) conjugated to Alexa 488 (green) following manufacture’s recommendations. The NOQ7.5.4 has been previously validated to identify fetal mouse myocytes and adult rabbit myocytes expressing the β-isoform (MHY7) of MyHC.[6]

### Statistics

Cells for volume analysis by Coulter Multisizer were collected in 3–6 replicates for each data point. FCM analysis of high light-scatter fractions was also collected in 3–6 replicates for each data point. Results are shown as mean±SD unless otherwise noted. The coefficient of variation (%CV) for a sample is indicative of assay imprecision. This is calculated by dividing the standard deviation of the observations by the mean of replicates. Cell volumes among 4 mesh groups were tested for significance with a one-way ANOVA followed by Tukey’s multiple comparison test. High-scatter cell fraction differences were tested with two-way ANOVA with individual comparison between mesh groups within in each animal. Two group comparisons were calculated by two-tail student t-test. Calculations were done using Prism 7 (GraphPad Software). Statistical significance was pre-specified at *p*<0.05.

## Results

### Animals

A four-month-old male rabbit (young, body weight (BW)- 2.87kg, heart weight (HW)- 8.6g) and six adult male rabbits (18-20-month-old, BW- 3.56±0.22kg, HW-10.05±0.98g) were studied. Ventricular myocyte HT was induced in two adult rabbits by a combined aortic valve insufficiency and abdominal aortic banding protocol that induces HT by chronic volume and pressure overload[18]. The other adult rabbits were age-matched controls (CNTL) without ventricular HT.

### Cell size bias with sample filtering

**Fig 1:**
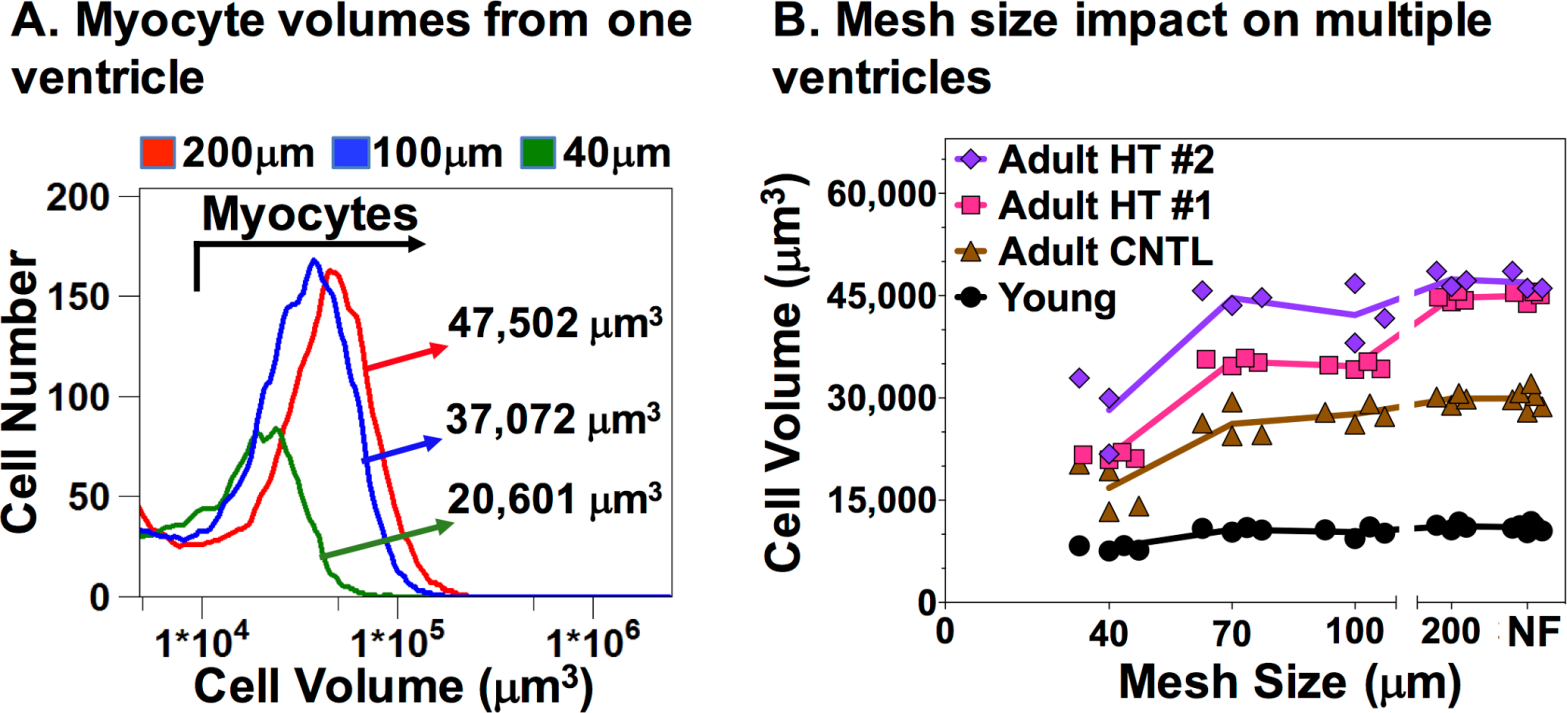
Mesh size and myocyte volumes. A, Ventricular myocytes (VMs) were obtained from an adult New Zealand White rabbit with cardiac HT (Adult HT#2). Three aliquots of cardiac cells in suspension were filtered through three different mesh sizes (40, 100, or 200μm) prior to sizing with a Coulter Multisizer. Cell volume distributions are plotted against cell numbers. The arrow in the plot shows the VM gate used for analysis; counts to the left are debris. 4000–7000 VMs were gated to calculate the median cell volume. Specific volumes for each mesh are noted in the plot. B, Myocyte volumes from four different rabbits (young, adult control (CNTL), and adults with cardiac hypertrophy (Adult HT #1 & 2)) were measured in replicates as shown in Panel A. Three to six aliquots from each ventricular sample were either not filtered (NF) or filtered through one of four mesh sizes (40, 70, 100, or 200μm) in multiple replicates. The mesh sizes are plotted on the x-axis starting with the NF samples (right to left). The median volumes of NF VMs increased substantially from young VMs (11,017+542μm^3^, 1.0-fold) to NF CNTL VMs (29,932+1,356μm^3^, 2.7-fold) to hypertrophic VMs (45,961+966μm^3^, 4.2-fold (n = 2). Each animal is color coded, and each mesh size has multiple replicates. Replicate data points are staggered at each mesh size to depict intra sample variance. Inflection points in the lines represent the mean value for each mesh within each animal sample.

Fig 1A shows the volume distribution of 4,000–7,000 VMs from a HT ventricle. We utilized a Coulter Multisizer to measure VM volumes in single-cell suspension because this technology is a robust assay that accurately measures cell volumes in VMs regardless of their shape[21, 22]. Before measuring the cell volume, aliquots of the parent cell preparation were filtered through different mesh sizes (i.e. 40, 100 or 200μm). The median VM volume filtered through a 200μm mesh was 47,502μm^3^. Filtering the same ventricular cell suspension through a 40μm mesh caused a 57% reduction in VM volume, and through a 100μm mesh caused a 22% reduction compared to the 200μm mesh.

To determine the extent of cell size biasing across a spectrum of VM sizes, we sampled three additional adult ventricles: one additional HT ventricle (i.e. large VMs), a young (i.e. small VMs) and an age-matched CNTL (i.e. intermediate size VMs). For these experiments, the volumes of filtered cells were compared to the volumes of cells collected without filtering (NF). Without filtering, the Coulter Multisizer clogged sporadically and those data points were discarded. No clogging was noted with filtered cells. Each sample had 3–6 aliquots filtered through each mesh of different sizes. As shown in Fig 1B, the median VM volume in the NF preparation increased from young to adult CNTL to HT VMs. However, the difference between NF VM volumes is diminished as the size of the mesh decreases. This artefactual cell size reduction is best depicted in the graph by a down sloping of each line when using a 100μm mesh or smaller.

**Fig 2:**
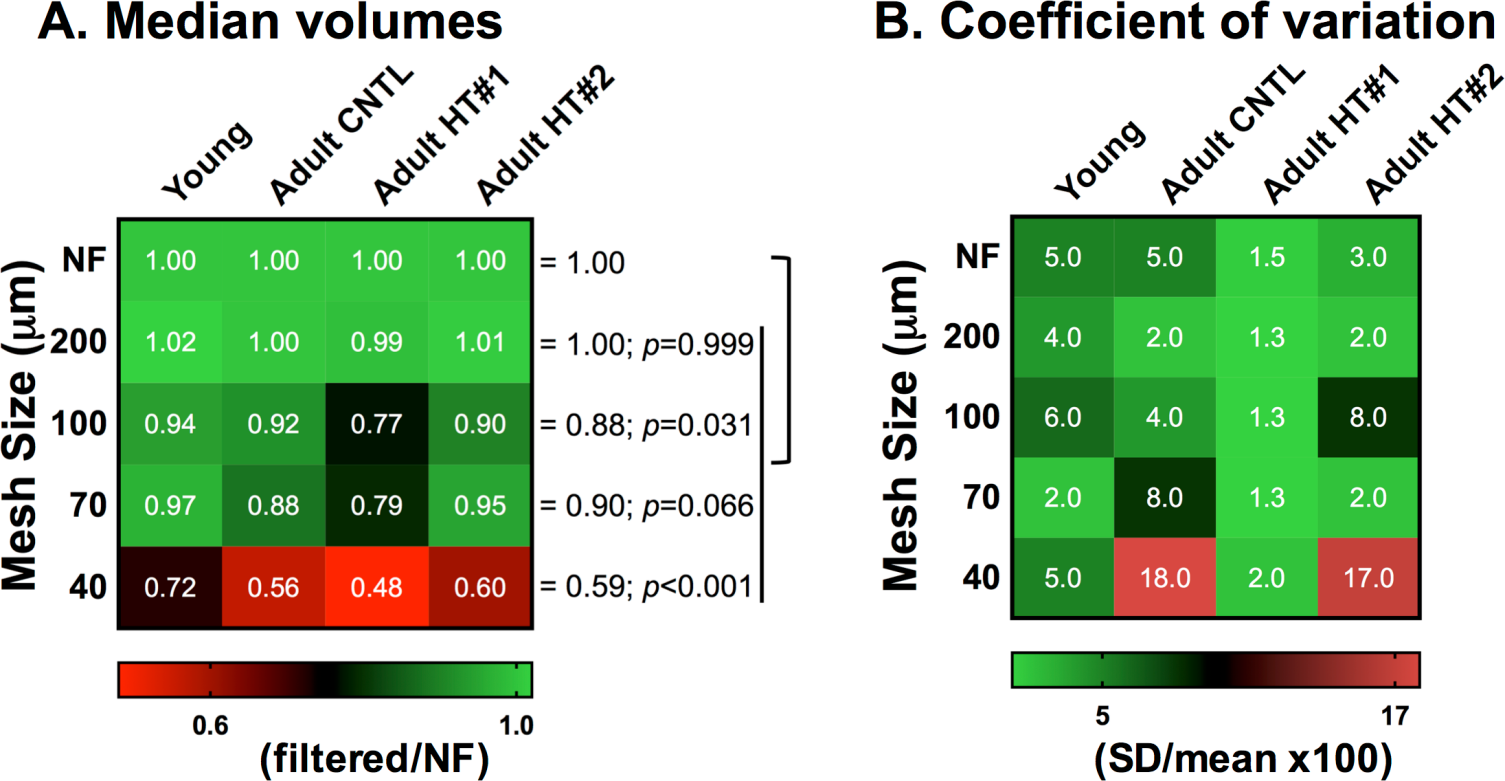
Variability in myocyte volumes. A, Myocyte volumes for filtered samples from each ventricle were normalized to the corresponding not-filtered (NF) volume (top row). Heat map values represent the fold change in median volume for each mesh size when compared to NF VMs. A 1.00 (lighter green) means no change in volume between the filtered and NF sample for each animal. The p values are calculated by one-way ANOVA among mesh size groups, and group to group comparisons between the NF VMs and each other mesh size were done with Tukey’s multiple comparison’s test. B, The coefficient of variation (%CV) is a measurement of imprecision that is calculated by dividing the standard deviation of identical replicates by the mean volume and multiplied by 100. The %CV is calculated for each ventricle per mesh using the replicates within sample (n = 3–6). A CV ≤5% (lighter green) is considered optimal for best assay reproducibility.

To determine how much variability any one mesh introduces into each preparation (i.e. four different rabbits), we calculated the fold change from the corresponding NF cells for each ventricle and across mesh size (Fig 2A). The NF cells provide the reference volumes and were treated as the “gold standard” for VM volume within each preparation. With the 200μm mesh, the average fold difference from the NF was 1±0.01 (n = 4). For the 40, 70 and 100μm meshes (n = 3–6) the fold differences were 0.59±0.09, 0.90±0.07 and 0.88±0.07 smaller, respectively (*p* = 0.031, *p* = 0.066, *p*<0.001, respectively).

The %CV provides an estimate of assay imprecision and is independent of the biological differences across preparations. Within each mesh size, NF and 200μm showed a %CV consistently below 5% for all four preparations (Fig 2B). However, the other mesh sizes had larger and more variable %CV (1.3–18%) indicative of a higher level of imprecision across ventricular preparations.

### Impact of washing and filtering on light-scatter profiles by FCM

**Fig 3:**
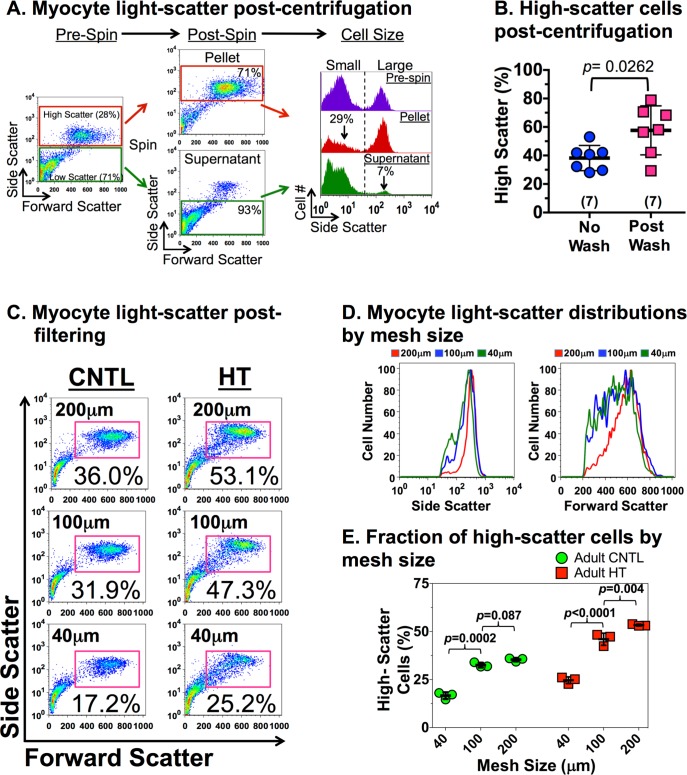
Variability in flow cytometric signatures. **A,** Ventricular cells were either not washed (pre-spin) or washed with low-speed centrifugation (post-spin, 50xg for 1 min x2), fixed and analyzed by flow cytometry (FCM). Post-spin, the pellet retains larger cells (i.e. ventricular myocytes, VMs) and the supernatant retains smaller cells (i.e. nonmyocytes). Cellular DNA is labeled with propidium iodide to gate on nucleated cells only. Bivariate plots show the forward and side-scatter signature of nucleated ventricular cells (4,000–7,000 cells per run). High-scatter (red box) and low-scatter (green box) sub-populations represent the predominantly VM and nonmyocyte subpopulations respectively, and are gated accordingly. Pre-and post-spin FCM show that the wash and spin loses large VM (~7%) to the supernatant and contaminates the pellet with small nonmyocytes (~29%). **B**, The fraction of high scatter cells were quantitated from multiple rabbits as shown in panel A without a wash or after a low-speed centrifugation. Number in parenthesis is the number of rabbits. The *p* values are calculated by two-tail student t-test. **C,** Ventricular cells from a control (CNTL) and an aged-matched rabbit with ventricular hypertrophy (HT) were fixed without a wash (to avoid losing any VM as shown above), and then filtered through one of three distinctive meshes (40, 100, or 200μm) prior to FCM analysis. The high-scatter sub-population noted in the pink gate contains the larger VMs. Percentages indicate the fraction of total nucleated cells in the sample that have a high-scatter signature after filtering. **D,** A set of FCM histograms for side scatter (left panel) and forward scatter (right panel) of HT high-scatter cells depict a leftward shift of cells as mesh size decreases. This shift is due to the relative oversampling of smaller cells than present in the parent preparation. **E,** The percentage of nucleated cells (3 replicates each) in the high-scatter gate are plotted for samples in panel B. Mesh size is noted in the x-axis. The *p* values are calculated by two-way ANOVA and individual group comparisons between mesh sizes within each rabbit.

**Fig 4:**
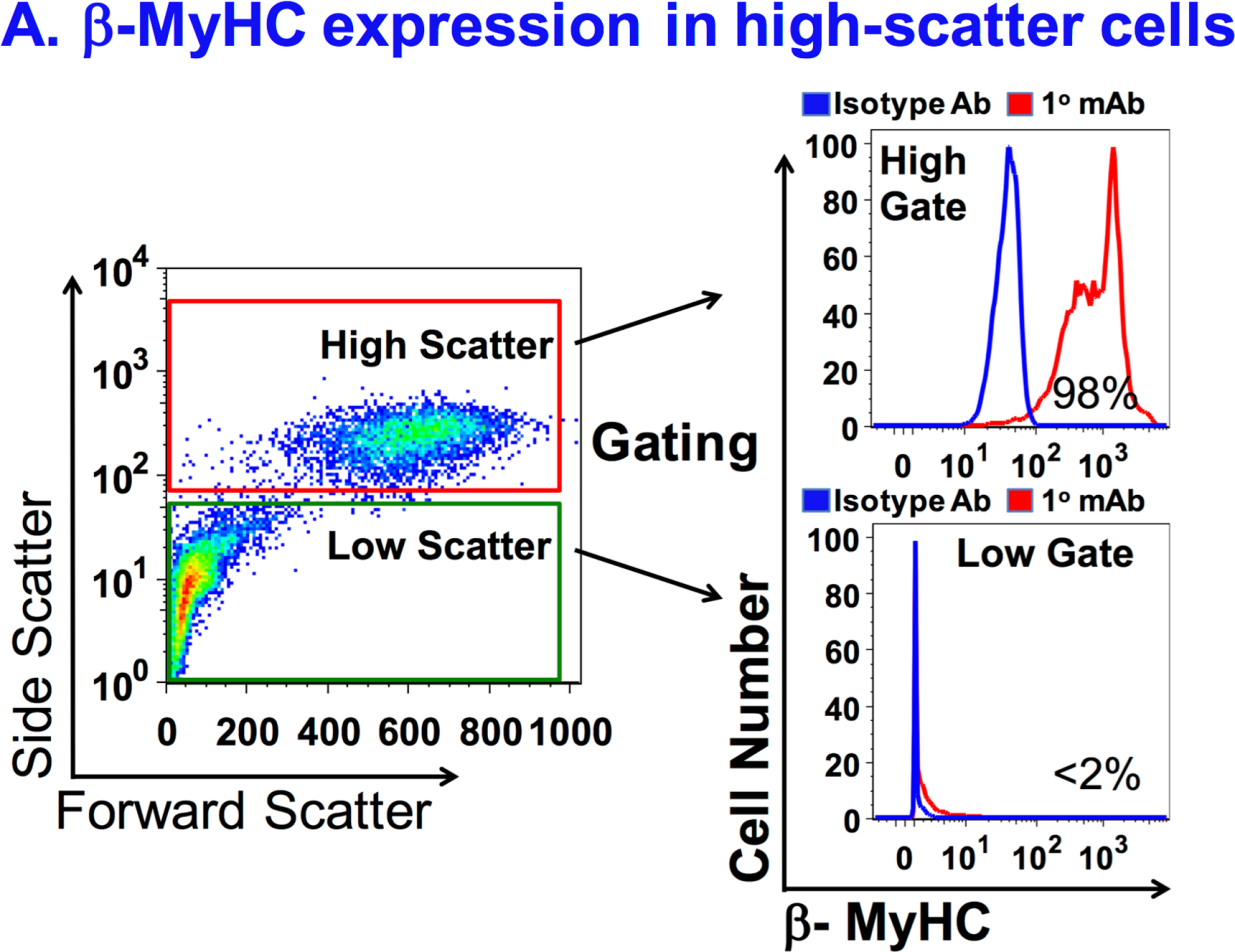
b-MyHC expression in high-scatter rabbit cells. A, Ventricular cells were prepared as described in Fig 3A. Bivariate plots show the forward and side-scatter signature of nucleated ventricular cells. High-scatter (red box) and low-scatter (green box) sub-populations are gated accordingly in histograms to the right. The cells were labeled with NOQ7.5.4D mAb to identify the expression of b-MyHC isoform. High scatter (top panel) and low scatter (bottom panel) cells in blue are stained with a non-specific IgG to determine background fluorescence. The cells in red are labeled with anti-b-MyHC mAb. The percent in each plot correspond to the fraction of ventricular myocytes in the analogous scatter gate.

Spun at low centrifugal forces, the resulting pellet of these ventricular preparations contained larger cells (i.e. myocytes) and the supernatant contained the smaller cells (i.e. nonmyocytes). Using a nucleated gate first, we utilized the high light-scatter signature to gate on VMs and determine the cell composition of these preparations[10]. VMs are usually a minor subpopulation of all cells by numbers while nonmyocytes are the major subpopulation.[6, 10, 12] The nonmyocytes are smaller in size and have a lower light-scatter profile (Fig 3A). We verified (Fig 4) that our high-scatter gate identifies the VM population to ~98% specificity by fluorescently staining samples with anti-β-MyHC that labels adult rabbit VMs.

To evaluate the impact of cell washes on cell composition, we quantitated the high-scatter cell fraction in pre-and post-spins samples (Fig 3A). The left panel shows bivariate FCM plot of nucleated cells before centrifugation (pre-spin). High scatter signature (28%, red gate) and low scatter signature (71%, green gate) change after two low-speed spins and washes. The post-spin suspension after washing contains 71% high-scatter cells and the supernatant contains 93% of low-scatter cells (middle panels). Cell sizes are plotted on histograms (right panel) demonstrating how the pellet has ~1/3 NVMs contaminants and the supernatant has ~7% of VMs which are now excluded from analysis in the pellet sample. The wash effect is quantitated in Fig 3B. The mean fraction of high-scatter cells (i.e. VMs) is 38±9% in the pre-wash, and consistent with prior findings in mice when samples are not washed[6, 12]. After wash, the mean fraction and variance (58±17%, n = 7, p = 0.026) are remarkably increased. This enrichment of VMs post wash is accompanied by an arbitrary loss of VMs in the supernatant collected after wash (Fig 3A, post-spin supernatant).

To determine the impact of filtering on VM light-scatter profiles, we compared cells from one CNTL to cells from a HT ventricle. Fig 3C shows bivariate plots from FCM after particles were gated for nucleated cells. The high-scatter subpopulation containing mostly larger VMs is gated (pink box) to quantitate their fraction over the nucleated cells. Because the cytometer can clog if samples are not filtered[6, 23], samples are compared to the cells filtered by the 200μm mesh. As seen with cell volumes by Coulter, the fraction of high scatter cells is reduced (Fig 3E) when cells are filtered with the 100 or 40μm mesh in the CNTL (8% and 53% respectively) and HT (14% and 54%) cells. A set of FCM histograms for side scatter and forward scatter (Fig 3D) depict a leftward shift of the HT high-scatter cells as mesh size decreases. This shift is due to the relative oversampling of smaller cells than present in the parent preparation. In summary, low-speed centrifugation arbitrarily increases the concentration of high-scatter VMs while filtering with smaller meshes arbitrarily reduces the numbers of larger VMs when compared to a sample with no wash or filtered through a 200μm mesh.

## Discussion

The main finding of this study is that cell washing and filtering can arbitrarily exclude VMs and change the composition of ventricular cells in single-cell preparations. This is critical when ventricular single-cell preparations are intended to represent all the cells in a heart during analysis. At the core of flow cytometric analysis is the principle that the sampled cells are representative of the cell composition of a parent population originally obtained from the tissue. If the sample is altered in any way during processing, the conclusions from the assay results are not applicable to all the cells from the parent population or tissue. The significance of our results is that adult VM preparations for FCM can be arbitrarily biased towards having smaller or higher concentration of myocytes than the parent population. These variations are most concerning when filtering and/or washing are not validated. If sampling arbitrarily smaller, fewer or more concentrated VMs than in the parent population is not the experimental design, our data demonstrates that results may be an experimental artifact.

### Lack of experimental standards for single-cell preparations from adult hearts

Research reproducibility is an emerging concern in biomedical research.[24–26] In a recent survey, more than 70% of researchers reported that they have tried and failed to reproduce another scientist’s experiments, and more than half have failed to reproduce their own experiments.[27] Reproducibility of results following multi-parameter FCM analysis has been controversial in other systems.[28] However, standardization in sample preparation, data acquisition, analysis strategy, and data presentation has provided reproducibility of results over time.[28, 29] Because adult VM FCM is still in early stages of implementation across laboratories, the time to establish experimental standards is prime.

However, there is no clear consensus on optimal protocols to isolate adult cardiac cells[1]. A comprehensive 2011 review of methods for VM isolations suggested that to enrich for viable VMs and remove undigested chunks of tissue, cell suspensions should be filtered through a 200 to 500μm mesh. They also suggested that when cells sediment either by gravity or gentle centrifugation, VMs can be separated from other cell types present in the parent population[2].

We reviewed fifteen reports (Table 1) from 1976 to 2017 that isolated cells from adult whole hearts (20%) or ventricles (80%). Five reports (33%) did not mention if cells were filtered. Three reports (20%) filtered cells with a 100μm mesh and the other seven reports (47%) filtered cells with meshes ≥ 200μm. Interestingly, two reports using 100μm meshes were recently published (2016 and 2017) as new methods for cardiovascular research[4, 30] suggesting a recent trend towards the use of smaller meshes when filtering adult VM preparations. A similar review of gentle centrifugation methods (Table 2) reflected that three reports (20%) used density gradients, and three other studies (20%) did not wash. Out of the ten studies that used gentle centrifugation, no two laboratories used the same protocol. Hence, the literature does not support the existence of a standard filtering or washing method for adult VM preparations.

**Table 1:** Ventricular myocyte isolations and cell filtering.

	Year	Species	Assay	Filtered	Mesh size (μm)
**Guo Y[4]**	2017	M	FCM	Yes	100
**López JE[10]**	2017	M	CV, FCM	Yes	200
**Ackers-Johnson M[30]**	2016	M	Micro	Yes	100
**Malliaras K[31]**	2013	R	FCM	Yes	250
**López JE[6]**	2011	M	CV, FCM	Yes	200
**Walsh S[32]**	2010	M	FCM	ND	NA
**Bergmann O[33]**	2009	H	FCM	Yes	100, 70
**Banerjee I[34]**	2007	M, R	FCM	ND	NA
**Strijdom H[35]**	2004	R	FCM	Yes	200
**Diez C[32]**	1998	R	FCM	Yes	250
**Armstrong SC[36]**	1992	R	FCM	ND	NA
**Campbell SE[21]**	1991	R	CV	Yes	250
**Gerdes AM[22]**	1986	R	CV	ND	NA
**Nash GB[14]**	1979	R	CV, FCM	ND	NA
**Powell T[37]**	1976	R	Micro	Yes	250

M = mouse, R = rabbit, H = Human, CV = Coulter volumes, FCM = flow cytometry, Micro = microscopy, ND = not described, NA = not applicable

**Table 2:** Ventricular myocyte isolations and cell washing.

	Year	Washed	Washing methods
**Guo Y[4]**	2017	Yes	20g x 4 min
**López JE[10]**	2017	No	NA
**Ackers-Johnson M[30]**	2016	Yes	300g x 5 min
**Malliaras K[31]**	2013	Yes	1x gravity
**López JE[6]**	2011	No	NA
**Walsh S[32]**	2010	Yes	ND
**Bergmann O[33]**	2009	Yes	Density gradient
**Banerjee I[34]**	2007	Yes	1,000g x 5 min
**Strijdom H[35]**	2004	Yes	100 RPM x 3 min, gravity x 5 min
**Diez C[32]**	1998	Yes	25g x 3 min
**Armstrong SC[36]**	1992	Yes	20g
**Campbell SE[21]**	1991	Yes	Density gradient
**Gerdes AM[22]**	1986	Yes	Density gradient
**Nash GB[14]**	1979	No	NA
**Powell T[37]**	1976	Yes	22g x 1min

ND = Not described, g = gravitational force, min = minutes, NA = not applicable, RPM = revolutions per minute

### Data reproducibility in adult ventricular myocyte flow cytometry

We found no significant difference in accuracy (Figs 1B and 2A) and precision (Fig 2B) when comparing the VM size of not-filtered sample to samples filtered with a 200μm mesh. This finding is true for VMs that range from volumes as low as 7,562μm^3^ (young heart) to as high as 48,595μm^3^ (HT#2 heart, Fig 1). We, however, did notice clogging of the Coulter Multisizer with not-filtered samples which prompted the need to eliminate the replicate runs with clogging. We had no clogging with samples filtered through a 200μm mesh and utilize this as the standard for further experiments.

In contrast, the smaller mesh sizes (≤100μm) led to artificially smaller VMs sizes than what was present in the non-filtered parent preparation (Figs 1 and 2). This change is presumably due to discarding larger VMs trapped in the mesh. In addition to the decreased accuracy on cell sizes, the imprecision of the cell size measurement was also increased (CV>5%, ranging from 1.3 to 18%, Fig 2). Together, these results support the optimal performance of the 200μm mesh in preparing VMs <50,000μm^3^ for FCM analysis without cell size biasing.

Because not-filtered preparation clogged our FACScan cytometer every time, we had to filter every FCM sample with the 200μm mesh. Since the cell volume analysis showed no biasing on the size of VMs filtered through the 200μm mesh, we filtered all samples for FCM accordingly. Cell washing with a low speed centrifugation is advocated to enhance the isolation of VM in cardiac preparations[2, 30]. Our comparison of washed and no-wash preparations (Fig 3A and 3B) demonstrated the arbitrary loss of VMs to the supernatant usually saved as the nonmyocyte fraction. In addition, ~1/3 of cells in the arbitrarily enriched VM fraction were still smaller nonmyocytes (Fig 3A). In conclusion, washing is an ineffective approach to enrich for VMs and it contributes to altering the composition of the parent preparation.

### Biological implications of cell size and composition biasing

The biological implication of arbitrarily changing adult VMs size and composition when filtering and washing preparations through a ≤100μm mesh is three-fold. First, the artefactual reduction of VM size is amplified as the VMs become larger. This is seen in our data where the largest absolute reduction in volumes occurs in the hypertrophy hearts filtered between the 100μm and the 200μm meshes (Fig 1B, HT#1 and HT#2 vs. CNTL and Young samples). This finding raises the concern that one laboratory may falsely minimize the effect size of cardiac injury or hypertrophy when VMs are filtered through a smaller filter than another laboratory.

Secondly, cardiac hypertrophy in other models of pressure overload like the trans aortic constriction model (TAC) in mice are known to vary between sub-populations of VMs in a single ventricle.[6] This observation was best documented by FCM analysis of all VMs from a single ventricle. In this model, VMs in the parent population are ~30–40% of all nucleated cells as seen in the current study with rabbit hearts. Of the two subpopulations in mice, the α-myosin heavy chain (MyHC) expressing VMs (~75% of VMs) after TAC hypertrophy by ~60%, whereas β-MyHC expressing VMs (~25% of VMs) only grow by ~10%. If one laboratory washes or filters samples after TAC differently to another, it is likely that VMs subtype composition and/or size would be experimentally different due to experimental artifacts.

Thirdly, adult VMs have a higher SSC and FSC light-scatter signal than nonmyocytes in FCM analysis because they are in the upward of 5–20 times larger. The higher VMs FCM signature shown in Fig 3C is consistent with our prior work in adult mice[6, 10] and that of Strijdom, et al.[35] in adult rats where samples were filtered through a 200μm mesh. In contrast Bergmann, et al.[33] and Guo, et al.[4] showed that adult VMs filtered with a 100μm had lower light-scatter signals that nearly equate the nonmyocytes in the preparation. Although they did not directly measure VMs size, these FCM signals suggest a technical artifact where only smaller VMs were sampled. Together, these experimental variations, if unintentional and/or unrecognized, can keep different laboratories from reporting similar results (i.e. diminished reproducibility) despite studying equivalent biological models.

### A proposed new standard for adult VM sample preparation

Our results identify sources of experimental variations when studying adult VMs for cell sizing and/or flow cytometry that have not been described previously. We provide data to assert that a no-wash step prior to cell fixation and filtration through a 200μm mesh would maintain the cell composition and VM size that actually exists in the adult heart when using cytometric analysis. This new finding could become part of the standard experimental design to minimize variable results that may not be reproducible across laboratories or a biological reality.

## Abbreviations

VM: ventricular myocyte
CV: coefficient of variation
FACS: fluorescent-activated cell sort
FCM: flow cytometric analysis
HT: hypertrophy
PFA: paraformaldehyde
